# Epidemiological Profile of Dental Trauma: A 13-Year Retrospective Study

**DOI:** 10.1155/ijod/1485407

**Published:** 2025-11-23

**Authors:** Samantha Jéssica Lopes Sousa, Liliana Vicente Melo De Lucas Rezende, Isla Maria Pereira Ibiapina, Ana Paula Dias Ribeiro, Maria Eduarda Casadei Motta Bellini, Daniella Birnbaum Pessoa De Mello, Júlio Cesar Franco Almeida, Fernanda Cristina Pimentel Garcia

**Affiliations:** ^1^Faculty of Health Sciences, Department of Dentistry, University of Brasília, Brasília, Federal District, Brazil; ^2^University of Florida, Gainesville, Florida, USA

**Keywords:** dental fractures, dental trauma, epidemiology, prevalence, tooth injuries

## Abstract

**Background/Aim:**

This study aimed to conduct a retrospective epidemiological investigation of patients treated in an extension project at a Brazilian dental school over the past 13 years.

**Material and Methods:**

Clinical records of patients treated at a university hospital in Brazil as part of a specialized dental trauma care project were reviewed. The study included both primary and permanent teeth and covered the period from 2011 to 2024. Statistical analysis was conducted using the Pearson chi-square, with a significance level set at 5%.

**Results:**

Of the 460 records evaluated, 375 met the inclusion criteria, encompassing a total of 833 affected teeth (220 primary and 613 permanent teeth). Males (*n* = 248) represented the majority of individuals treated and exhibited a higher prevalence of hard tissue injuries (*n* = 208) compared with females (*n* = 93). The most common type of hard tissue injury was enamel and dentin fractures without pulp exposure (*n* = 139). Patients with hard tissue injuries generally sought care promptly after the traumatic event (*p* < 0.0001) and showed a significantly higher incidence of endodontic treatment needs (*p* < 0.0001) than those soft tissue fractures. Falls were identified as the leading cause of all types of hard tissue fractures (*p* < 0.0001).

**Conclusions:**

The study identifies a high-risk profile for hard issue injuries, predominantly affecting children from infancy to early adolescence (ages 0–14 years), with falls being the most frequent cause. Additionally, hard tissue injuries were associated with faster care-seeking behavior and a higher likelihood of requiring endodontic treatment.

## 1. Introduction

Dental trauma can occur across all age groups but is more commonly seen in children and young adults. Approximately 25% of children and 33% of adults experience trauma to the permanent dentition. Across all age groups, men are more prone to dental injuries than women [1]. It is estimated that over 1 billion people currently alive have suffered some form of dental trauma according to studies from the last 6 years [2].

The International Association of Dental Traumatology (IADT) (2020) classifies dental injuries involving hard tissues (fractures) into various categories: enamel infractions (not considered a complete fracture), enamel fractures, enamel and dentin fractures, enamel and dentin fractures with pulp exposure, crown–root fractures without pulp exposure, crown–root fractures with pulp exposure, root fractures, and alveolar fractures [3]. Even fractures limited to the coronal portion of the tooth, without pulpal, radicular, or bony involvement can have a highly variable prognosis depending on the patient's profile and specific characteristics. Whether treated or not, fractures extending beyond the enamel negatively impact children's quality of life, affecting them socially and emotionally [4].

Recent studies indicate an increased tendency to seek immediate care when visible or more severe trauma, particularly involving hard tissues, is present [5]. This clinical and epidemiological profile emphasizes the importance of understanding the presentation of these injuries and the demographic characteristics of affected patients [6]. Knowledge of the epidemiology and patient distribution in cases of dental trauma provides a foundation for developing clinical protocols, first-aid guidelines, and methods of documenting and evaluating traumatic dental injuries [6, 7].

Therefore, this study aimed to conduct a 13-year retrospective epidemiological investigation of dental trauma in a university extension project, focusing on both primary and permanent teeth, with special attention to the prevalence and patterns of hard tissue injuries, their associated risk factors, and treatment needs.

## 2. Material and Methods

This study was approved by the research ethics committee (CAAE: 37555320.7.0000.0030), followed the guidelines updated by the IADT in 2020, and adhered to the guidelines outlined in the Preferred Reporting Items for Observational Studies in Endodontics (PROBE) 2023 [8]. All patients included in the study had consent forms signed by their legal guardians or by themselves, as appropriate. This was a longitudinal observational study that retrospectively analyzed patient records from the Dental Trauma Extension Project (DTEP) at the University Hospital of Brasília over 13 years (2011–2024). Data were collected using a structured form developed in Google Forms, where information from the medical records was transcribed according to predefined independent variables based on the existing literature. These records were used to generate Excel tables and individual graphs showing the prevalence of each independent variable.

The study included the medical records of all patients treated at the DTEP at the University of Brasília from 2011 to 2024. Patients were included if their legal guardians had signed the informed consent form in all clinical records. Records that lacked complete documentation or were missing were excluded from the analysis. Patient documentation comprised clinical records containing medical and dental history, trauma-specific data, and photographic and radiographic documentation. Follow-up care was based on IADT guidelines (2020), with adjustments according to the type and severity of the trauma.

The collected data included sex (male and female), age, etiology (fall, collision, and automotive or cycling accident), type of trauma [3, 9], affected tooth, number of affected teeth, location where the trauma occurred (home, school, street, and other), time elapsed since trauma (within 12 h, 12–24 h, or days), treatment performed (extraction, endodontic treatment, restoration/prosthesis, and others), clinical sequelae (no sequelae, color change, fistula or edema, ectopic position, or premature loss), radiographic sequelae (no sequelae, pathological root resorption, accelerated root resorption, pulpal canal obliteration, and periapical radiolucency), and all other available information in the patient records.

Before the data collection phase, the researcher responsible for the analysis conducted a pilot study on 10 patient records under the supervision of a professor to standardize the data collection process. Weekly theoretical sessions were held, and the researcher also participated in clinical activities as a permanent member of the DTEP to reinforce these concepts. A Google Form was created to systematically register the data from each patient's clinical history as part of the extension project, resulting in a digital version of the findings in the patient's records. The data were converted into Excel spreadsheets and analyzed using Stata version 11.0. Descriptive statistics were used to analyze qualitative variables through absolute and relative values.

To assess associations between independent variables and outcomes, the Pearson chi-squared test was used with a significance level of 5% (*α* = 0.05). After the statistical analysis, the highest-risk group, based on factors such as age, gender, causes, and trauma location, was identified for a subsequent phase of this project, which will involve a qualitative evaluation of these patients to propose preventive and health education measures.

## 3. Results

Between 2011 and 2024, 460 patients were seen by the project. Of these, 24 were excluded due to noncompliance with the inclusion criteria (e.g., lack of consent for data use or lost records). Thus, 436 records were analyzed, focusing on trauma incidence across various independent variables, including gender, etiology, type of trauma, affected tooth, trauma location, time elapsed since trauma, and treatments performed. Among these patients, 61 (13.8%) were seen for preventive measures, specifically for the creation of sport mouthguards, without any history of dental trauma. Therefore, between August 2011 and August 2024, 375 patients were treated for dental trauma, involving a total of 833 teeth (220 primary and 613 permanent teeth) as shown in Tables 1, 2, and 3.

Table 1 shows the distribution of hard tissue involvement according to gender and age. The chi-square test revealed no significant difference regarding gender (*p* > 0.05), but age was significantly associated with the presence of hard tissue injuries (*p* < 0.05).

Table 2 presents the association between etiology and hard tissue involvement. Falls were the most frequent cause, followed by collisions and traffic accidents. There was a statistically significant difference between groups for etiology (*p* < 0.05), although some categories such as “Others” and “Not registered” had very low frequencies, which may affect the validity of the chi-square test.

Table 3 shows the association between time to first-aid, need for endodontic treatment, and hard tissue involvement. A significant difference was observed for both variables (*p* < 0.05). Patients with hard tissue injuries tended to seek care sooner and had a higher demand for endodontic treatment compared with those soft tissue involvement.

Overall, the Pearson chi-squared test indicated sufficient statistical evidence to reject the null hypothesis for the variables age, etiology, time elapsed until first treatment, and the need for endodontic treatment, while no significant differences were found for gender. It is important to note that statistically significant associations do not necessarily imply causation.

**Figure 1:**
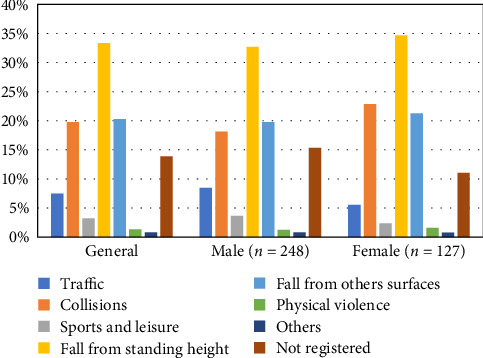
Etiological factors for the occurrence of dental trauma, general, and according to gender.

Of the patients treated, 65.8% were male. In the primary dentition, the most commonly affected tooth was the left maxillary central incisor (37.3%), while, in the permanent dentition, the right maxillary central incisor was most frequently involved (33.4%). Regarding the etiology, falls from standing height accounted for 33.3% of cases, followed by falls from other surfaces or objects (20.3%), collisions (19.7%), and traffic accidents (7.5%) (Figure 1). The “Other” category included workplace accidents and injuries sustained during orotracheal intubation.

**Figure 2:**
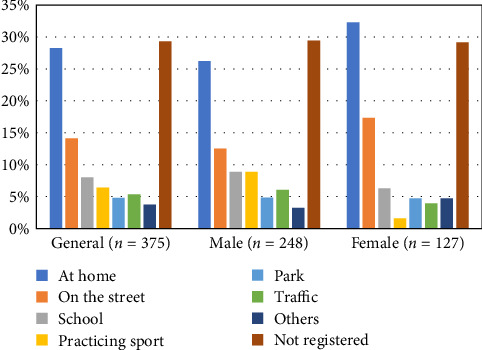
Location of occurrence of dental trauma, general, and according to gender.

Only 25.6% of cases (*n* = 96) received care within the week of the trauma. The most common locations where the trauma occurred were at home (28.3%), on the street (14.1%), and in school settings (8%) (Figure 2).

Clinical symptoms were reported in 120 of the 375 trauma patients (32%). The most frequently observed symptoms were pain during mastication (16%), spontaneous toothache (10.67%), thermal sensitivity (10.13%), and loss of consciousness (5.87%). Less common symptoms included nausea or vomiting (4.8%), disorientation (3.47%), neck pain (2.13%), hemorrhage (2.13%), and dizziness (0.53%). Extraoral complications were observed in 27.7% of cases, including swelling (13.1%), abrasions (6.9%), bruises (6.13%), external hemorrhages (3.2%), facial bone fractures (1.86%), and the presence of foreign bodies (0.26%). Intraoral injuries were observed in 20.53% of cases and included lip injuries (7.73%), periodontal damage (4.53%), oral mucosal lesions (3.73%), frenulum injuries (2.13%), tongue injuries (0.53%), and palate injuries (0.27%). Additionally, increased overjet was recorded in 4.53% of cases, increased overbite in 4.27%, and crossbite in 1.6%.

Common clinical findings included tooth mobility (14.13%), a positive response to thermal testing (12.4%), tooth discoloration (12%), percussion pain (8%), and unsatisfactory restorations or caries in the affected teeth (2.67%). Radiographic findings showed periapical lesions (6.93%), widening of the periodontal space (5.33%), pathological internal or external resorption (2.67%), and pulp chamber calcification (2.4%).

Hard tissue injuries predominantly involved enamel and dentin fractures without pulp exposure (46.3%), followed by enamel fractures (18.3%) and crown fractures with pulp exposure (17.3%). Notably, 38.93% of the records lacked information on hard tissue trauma. Regarding soft tissue injuries, of the 375 patients with dental trauma, 171 were classified as having soft tissue injuries. Among these, dental avulsion (31.9%), lateral luxation (27.3%), and concussion or subluxation (19.5%) were the most common, while 54.4% of records did not indicate soft tissue involvement (Table 4). Regarding dental avulsion cases, 21.62% of teeth were transported to the clinic in a dry environment (e.g., paper, napkin, and pocket), while only 10.8% of patients used a hydrated medium (e.g., saline, milk, and water). In 28.38% of cases, patients sought care within 24 h of the avulsion, with 4.05% presenting with immediate reimplantation and 4.05% having lost or discarded the avulsed tooth.

**Figure 3:**
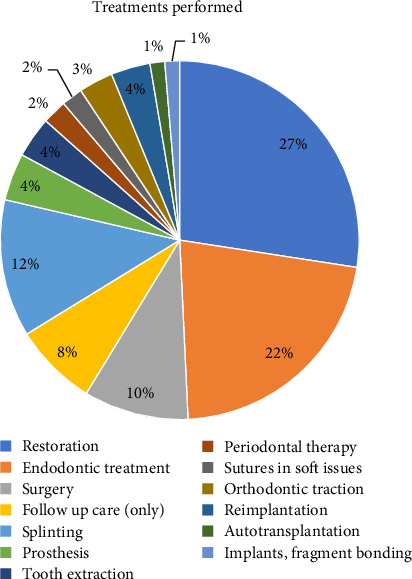
Treatment performed.

According to Table 4, the Pearson chi-squared hypothesis test rejected the null hypothesis for the categorical variables age, time elapsed until first treatment, and need for endodontic treatment, considering patients affected by dental trauma involving all types of hard tissue fractures presented. There is sufficient statistical evidence to indicate significant differences between the groups in the variables age (*p* < 0.05), etiology (*p* < 0.0001), time elapsed until first treatment (*p* < 0.0001), and the need for endodontic treatment (*p* < 0.0001). It can be categorically stated that there is a difference in the occurrence of these types of injuries according to each variable. However, with respect to gender in relation to the different types of dental trauma injuries, there was insufficient statistical evidence to reject the null hypothesis, as per the gender variable (*p*  > 0.05), indicating that the occurrence of different types of hard tissue fractures does not vary significantly between men and women. The treatments performed on the patients evaluated are presented in Figure 3.

**Figure 4:**
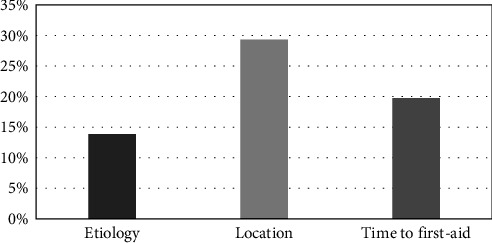
Unreported or unrecovered information on the etiology, location, and time elapsed between dental trauma and first care.

A notable concern was the high prevalence of incomplete information in the records. Approximately, 19.73% of patients were unable to recall or report the time elapsed since trauma, 13.86% were unsure how the trauma occurred, and 29.33% of records lacked information on the trauma location (Figure 4).

## 4. Discussion

Traumatic dental injuries affected predominantly children aged 0–14 years, a finding consistent with the vulnerability of this group due to immature motor skills and increased exposure to falls. Enamel–dentin fractures were the most common diagnosis (46.3%), and less than one quarter of patients received care within the first week. This delay in treatment is known to negatively influence prognosis and has also been reported in other epidemiological studies, although differences in trauma etiology were observed when compared with other regions, where traffic accidents are more frequent [10–13].

The IADT provides age-specific prevention guidelines, emphasizing home safety in infants, parental counseling in toddlers, and reinforcement of protective measures and dental checkups during childhood and adolescence.

Gender differences in trauma incidence were evident, with boys comprising 66.79% of those under 14 years. Many studies attribute this to boys engaging in riskier play [14–16]. However, other studies suggest that age, location, and type of fall or dental phase are more relevant factors than gender [17–20].

Maxillary central incisors were the most affected teeth, consistent with literature due to their exposed position and reduced lip protection. Falls, bicycle accidents, collisions, and automobile accidents were the main etiologies, reinforcing the importance of preventive measures and public education campaigns. The IADT emphasizes the role of protective devices in sports and public education campaigns.

Incomplete anamnesis was observed in up to 29% of records, limiting diagnostic accuracy. Only 32% of patients presented clinical symptoms, likely due to delayed care, which complicates prognosis. Nearly half required restorative treatment and 39.5% endodontic procedures, in agreement with IADT recommendations [21, 22].

A study by the Endodontic Clinic at the University of São Paulo's School of Dentistry identified a high prevalence of enamel and dentin fractures, a finding consistent with the data from the IADT [3, 9, 13, 21, 23]. The occurrence of multiple injuries in the same tooth is unfavorable for the patient's prognosis [24]. Crown fractures significantly increase the risk of pulp necrosis and infection in teeth with concussion or subluxation, especially when root formation is complete [21]. Likewise, crown fractures with or without pulp exposure raise the risk of pulp necrosis and infection in teeth with lateral luxation. The IADT guidelines [3, 9, 13, 23] provide specific treatment recommendations and prognosis for each traumatic injury, guiding professionals and specialists toward proper management for the best possible outcomes. In this study, due to the high prevalence of enamel and dentin fractures without pulp exposure, 49.6% of treatments involved restorations, followed by 39.46% endodontic procedures.

Community education on avulsion management is essential, as prognosis depends on immediate first-aid (cleaning, moist storage, crown handling, and prompt dental care). In our sample, avulsion exceeded 30%, reinforcing the need for public awareness and preventive guidance. Dental professionals must act as educators, especially for high-risk groups, by promoting preventive measures and the use of protective equipment. First aid involves hemorrhage control, risk assessment, and evaluation of fractures or luxations, and this knowledge should be disseminated among all healthcare professionals to improve outcomes [25–27].

A major limitation was the high prevalence of incomplete records, with up to 29% missing essential information such as etiology, time, or location of trauma. This restricts epidemiological accuracy and clinical decision-making, underscoring the need to improve standardized protocols in trauma centers and to reinforce preventive education in the community.

## 5. Conclusion

This 13-year retrospective study demonstrates that dental trauma predominantly affects children aged 0–14 years, with falls being the main etiology. A high prevalence of enamel–dentin fractures was observed, frequently requiring restorative treatment and, in some cases, endodontic therapy, highlighting the clinical impact of these injuries. A considerable proportion of patients sought care only after critical time intervals, compromising prognosis. Additionally, the high rate of incomplete documentation impaired epidemiological accuracy and clinical decision-making. These findings emphasize the urgent need to strengthen record-keeping protocols, ensure early access to care, and implement preventive strategies tailored to the most vulnerable groups.

## Figures and Tables

**Table 1:** Association between gender and age with hard tissue involvement.

Variable	With hard tissue involvement (*n* = 229)	Soft tissue injuries (*n* = 146)	Chi-square	*p*-Value
Gender				
Male	65.93% (*n* = 151)	66.44% (*n* = 97)	0.0099	0.9206
Female	34.07% (*n* = 78)	33.56% (*n* = 49)	—	—
Age				
<14 years old	143	104	—	—
14–25 years old	42	14	6.6854	0.0353^*∗*^
≥26 years old	44	21	—	—
Not registered	0	7	—	—

*Note:⁣* ^*∗*^ indicates statistically significant difference.

**Table 2:** Association between etiology and hard tissue involvement.

Etiology	With hard tissue involvement (*n* = 229)	Soft tissue injuries (*n* = 146)	Chi-square	*p*-Value
Traffic	19	9	—	—
Sport and leisure	8	4	—	—
Collisions	44	30	18.399	0.0053^*∗*^
Fall	132	69	—	—
Physical violence	4	1	—	—
Others	3	0	—	—
Not registered	19	33	—	—

*Note:⁣* ^*∗*^ indicates statistically significant difference.

**Table 3:** Association between time to first-aid, need for endodontic treatment, and hard tissue involvement.

Variable	With hard tissue involvement (*n* = 229)	Soft tissue injuries (*n* = 146)	Chi-square	*p*-Value
Time from trauma to first-aid				
≤7 days	56	40	—	—
8–30 days	51	24	—	—
1–3 months	33	12	22.39	0.0021^*∗*^
4–6 months	23	6	—	—
7–11 months	5	3	—	—
1–3 years	19	9	—	—
≥3 years	12	8	—	—
Not registered	30	44	—	—
Need for endodontic treatment				
Yes	109	33	—	—
No	114	84	44.1268	<0.0001^*∗*^
Not registered	6	29	—	—

*Note:⁣* ^*∗*^ indicates statistically significant difference.

**Table 4:** Association of types of hard tissue trauma injuries (enamel fracture, enamel–dentin fracture, complicated crown fracture, crown–root fracture, root fracture, and alveolar fracture) with gender, age, etiology, time elapsed until first treatment, and need for endodontic treatment.

Variable	Enamel fracture (*n* = 56)	Enamel–dentin fracture (*n* = 139)	Complicated crown fracture (*n* = 52)	Crown–root fracture (*n* = 31)	Root fracture (*n* = 17)	Alveolar fracture (*n* = 6)	Chi- square	*p*-Value
Gender								
Male	37	101	35	22	9	4	3.292	0.0696
Female	19	38	17	9	8	2		
Age								
<14 years old	36	85	26	14	13	3	10.401	0.0013^*∗*^
14–25 years old	11	23	12	7	2	0		—
≥26 years old	9	31	14	9	2	3		—
Etiology								
Traffic	6	15	3	2	5	1	29,157	<0.0001^*∗*^
Sport and leisure	1	6	2	0	1	1		
Collisions	10	25	13	6	1	1		
Fall	33	67	24	15	9	3		
Physical violence	0	4	1	1	0	0		
Others	1	0	1	2	0	0		
Not registered	6	10	3	5	1	0		
Time from dental traumatic injuries to first-aid								
≤7 days	18	33	17	8	5	0	40,980	<0.0001^*∗*^
8–30 days	10	32	11	5	2	5		
1–3 months	9	19	5	3	1	1		
4–6 months	6	12	6	3	4	0		
7–11 months	0	4	2	0	0	0		
1–3 years	4	9	4	6	2	0		
≥3 years	1	10	0	0	1	0		
Not registered	8	19	7	5	2	0		
Need for endodontic treatment								
Yes	22	64	44	19	9	4	36,695	<0.0001^*∗*^
No	35	72	7	9	7	2		
Not registered	0	4	1	2	1	0		

*Note:⁣* ^*∗*^ indicates statistically significant difference.

## Data Availability

The data that support the findings of this study are available upon request from the corresponding author. The data are not publicly available due to privacy or ethical restrictions.
